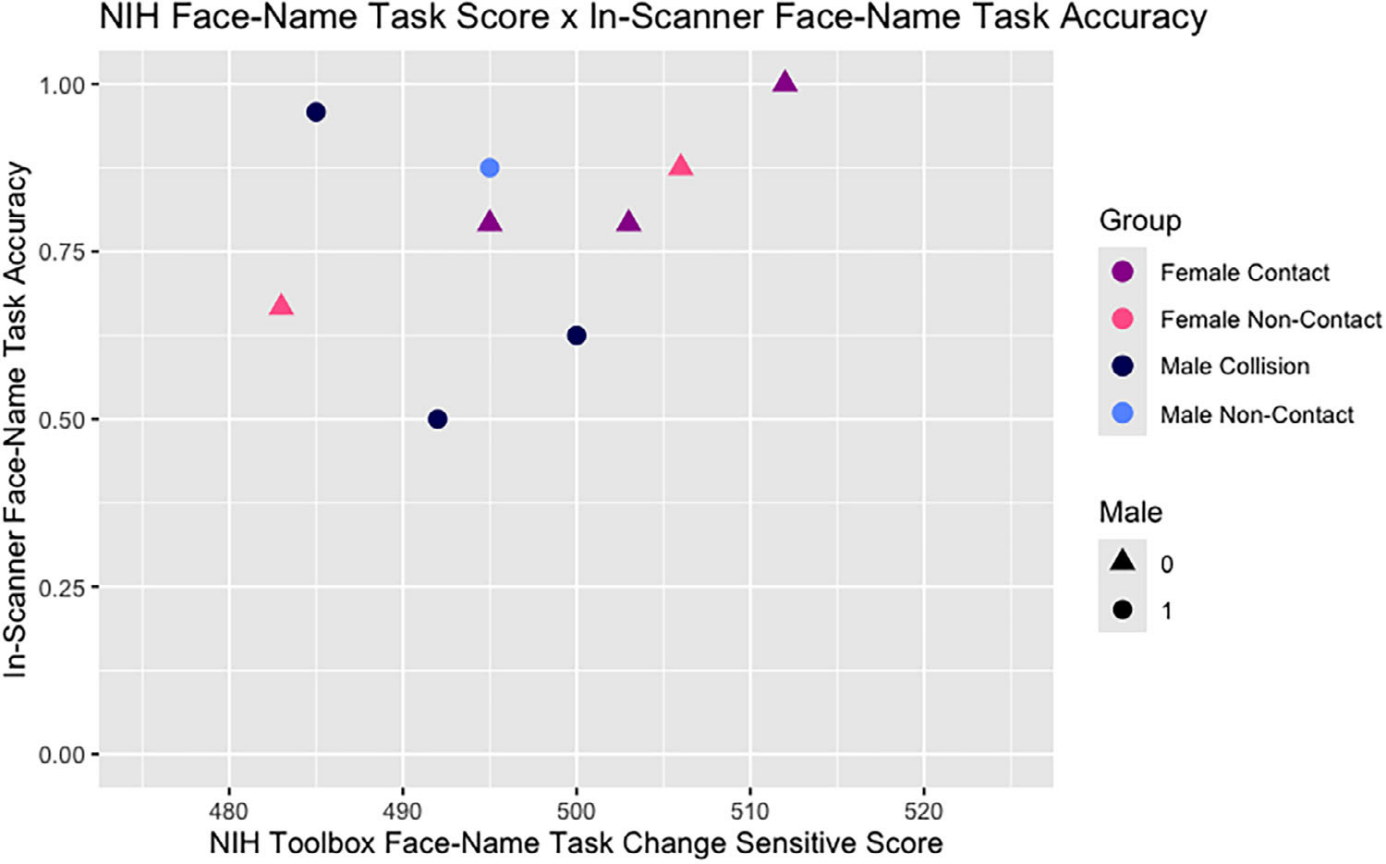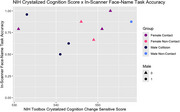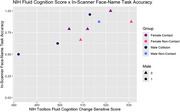# Exploring Episodic Memory in Older Former High School Athletes with Preliminary Findings from an Inclusive Face‐Name fMRI Task

**DOI:** 10.1002/alz70861_108432

**Published:** 2025-12-23

**Authors:** Michaela Broadnax, Julia Leskow, Philip Veliz, Katharine Seagly, James T Eckner, Eleanna Varangis

**Affiliations:** ^1^ University of Michigan School of Kinesiology, Ann Arbor, MI USA; ^2^ Michigan Concussion Center, Ann Arbor, MI USA; ^3^ University of Michigan School of Nursing, Ann Arbor, MI USA; ^4^ University of Michigan Medicine Physical Medicine & Rehabilitation, Ann Arbor, MI USA

## Abstract

**Background:**

Older adults with a history of sport‐related concussions and repetitive head impacts report increased difficulty forming new episodic memories. A common task of episodic memory in older adults is a face‐name memory task, however current versions of the task include faces that are not always representative of retired athlete populations, and frequently include faces of predominantly younger adults. The present study shows preliminary feasibility data for a novel inclusive face‐name memory task developed specifically for use in the context of an fMRI scan.

**Methods:**

We created a racially inclusive face‐name memory task tailored for middle‐aged and older adults (age 50‐80; target n=100) for use in an MRI scanner. The task is comprised of 2 6‐minute runs, each including 3 blocks of encoding and retrieval phases, and featuring 4 face‐name pairs per block representing diverse racial and ethnic backgrounds. Participants perform the task during an fMRI neuroimaging session to evaluate both behavioral performance on the task as well as the neural correlates of memory encoding and retrieval. Participants in the present study included men (*n* =4) and women (*n* =5) who had participated in at least 2 years of sport in High School, but ceased participation in organized sport after High School (ages 51‐77). Sport participation was classified as collision (*n* =3) vs. non‐contact (*n* =1) sport for male participants, and contact/collision (*n* =3) vs. non‐contact (*n* =2) sport for female participants.

**Results:**

Preliminary behavioral data from the first 9 participants indicate that participants generally perform quite well on the in‐scanner task (ranging from 50‐100% accuracy; mean = 78.7%), and that overall task performance is correlated with Fluid (r=0.839; *p* =0.005), but not Crystalized (r=0.156; *p* =0.688) cognition (NIH Toolbox Cognition Battery), and while not significantly correlated, it seems to generally track with a face‐name memory task included as part of the NIH Toolbox battery (r=0.355; *p* =0.349).

**Conclusions:**

Preliminary data suggest that our novel in‐scanner face‐name episodic memory task is feasible for older former High School athletes to perform in an MRI scanner, and that performance seems to track with other standardized assessments of Fluid cognition.